# Case of Worms Discharged through a Wound of the Groin

**Published:** 1786

**Authors:** William Coleman

**Affiliations:** Surgeon at Sandwich in Kent


### III. *Cafe of Worms difcharged through a Wound of the Groin. Communicated in a Letter to Dr.* Simmons, *F. R. S. by Mr.* William Coleman, *Surgeon at Sandwich in Kent.*

HAVING lately had a cafe under my care which feems to be a remarkable one, I take the liberty of fending you the following account of it, to be inferted, if you think proper, in the London Medical Journal:

Elizabeth Dawkins, a poor married woman, aged forty years, of the parifh of Word, near Sandwich, was feized, on the 12th of December, 1785, with a violent pain of her bowels, and vomiting, for which my affiftance was defired. On examination, I difcovered a fwelling in the right groin, and was told, that, for feveral years paft, fhe had been troubled with a hernia, which had come on after a difficult labour; but which, till now, fhe had been always able to reduce herfelf.

Suppofing her complaints to be occafioned by a fmall portion of the inteftine having flipt through the ring, I endeavoured to reduce it, and fucceeded, fo that no tumour remained.

As ſhe had had no ſtool for ſeveral days, an active purgative was adminiſtered; but this being rejected by vomiting, and the pain continuing with great ſeverity, a purgative clyſter was injected, which procured a copious evacuation by ſtool, ſo that I now flattered myſelf all danger was over.

When I viſited her again on the 14th, I was ſurpriſed to find a large ſwelling in the groin, in ſuch a ſtate of inflammation as clearly indicated that matter was forming. To this tumour a poultice of white bread and milk was directed to be applied, and on the 16th the patient ſent me word that the ſwelling was burſt.

When I came to her, I found the part ſphacelated to the extent of three inches in length, and one and a half in breadth. The ſphacelated part being already in a great meaſure ſeparated from the ſound, as much of it as conveniently could be was removed, to give a free diſcharge to the matter, and I was greatly aſtoniſhed to find in the wound a round worm (lumbricus) of about eight inches in length, and dead. The day following, on removing the dreſſings, I found one worm alive. On the 18th and 19th, the cavity of the wound was almoſt filled with them, as on each of thoſe days I found in it three live worms, all of them eight or nine inches in length. A ſingle live worm was diſcharged in the ſame manner on the 21ſt, and another about ſix days after.

On the 6th of January, 1786, ſhe took twelve grains of jalap in powder, and ſix grains of calomel, which brought away two dead worms by ſtool. Two live ones paſſed the ſame day through the wound.

January 12, the mercurial purge was repeated; and, on taking off the dreſſings that day, part of a live worm appeared in the wound, making its way from under Pouparts ligament. This I drew out with the forceps.

The wound was now in a great meaſure healed, and I did not ſee her again till January the 23d. In the interim ſhe had taken, by my direction, *pulv. ſtann.* ʒj. every morning, and a tea-cupful of a ſtrong decoction of Indian pink every night, and had voided three live worms through the wound, and two dead ones by ſtool. Since that time ſhe has been apparently in good health; but a fiſtulous ſore ſtill remains in the groin, through which a purulent matter, ſlightly tinged with fæces, and, and, from time to time, a round worm conti-nue to be difcharged *.

*Sandwich,*
July 5, 1786.

* The reader will find a cafe fimilar to this in the firft volume of the Edinburgh Medical Effays, art. xix.

